# Supplementary material for: Molecular Mimicry Between Toxoplasma gondii B-Cell Epitopes and Human Antigens Related to Schizophrenia: An In Silico Approach
Source: Int J Mol Sci. 2025 Oct 23;26(21):10321. doi: 10.3390/ijms262110321 (PMC12609498; doi:10.3390/ijms262110321)
Supplement: Supplementary file 1 [file ijms-26-10321-s001.zip › ijms-3902395-supplementary.pdf]

Supplementary Material

Supplementary Table S1: Autoantigens of schizophrenia retrieved by AAg Atlas and literature review.

#	Human Autoantigen Gene	Protein	Biological function	Acc. Number	Molecular weight (Da)	Length (Amino acids)
1	*ENO2*	Gamma-enolase	Has neurotrophic and neuroprotective properties on a broad spectrum of central nervous system (CNS) neurons. Binds, in a calcium-dependent manner, to cultured neocortical neurons and promotes cell survival (By similarity).	P09104	47,269	434
2	*CHRM1*	Muscarinic acetylcholine receptor M1	The muscarinic acetylcholine receptor mediates various cellular responses, including inhibition of adenylate cyclase, breakdown of phosphoinositides and modulation of potassium channels through the action of G proteins. Primary transducing effect is Pi turnover.	P11229	51,421	460
3	*ELANE*	Neutrophil elastase	Serine protease that modifies the functions of natural killer cells, monocytes and granulocytes. Inhibits C5a-dependent neutrophil enzyme release and chemotaxis (PubMed:15140022). Promotes cleavage of GSDMB, thereby inhibiting pyroptosis (PubMed:36899106). Capable of killing E.coli but not S.aureus in vitro; digests outer membrane protein A (ompA) in E.coli and K.pneumoniae (PubMed:10947984).	P08246	28,518	267

#	Human Autoantigen Gene	Protein	Biological function	Acc. Number	Molecular weight (Da)	Length (Amino acids)
4	*IL2*	Interleukin-2	Cytokine produced by activated CD4-positive helper T-cells and to a lesser extend activated CD8-positive T-cells and natural killer (NK) cells that plays pivotal roles in the immune response and tolerance (PubMed:6438535).<br>Binds to a receptor complex composed of either the high-affinity trimeric IL-2R (IL2RA/CD25, IL2RB/CD122 and IL2RG/CD132) or the low-affinity dimeric IL-2R (IL2RB and IL2RG) (PubMed:16293754, PubMed:16477002).<br>Interaction with the receptor leads to oligomerization and conformation changes in the IL-2R subunits resulting in downstream signaling starting with phosphorylation of JAK1 and JAK3 (PubMed:7973659).<br>In turn, JAK1 and JAK3 phosphorylate the receptor to form a docking site leading to the phosphorylation of several substrates including STAT5 (PubMed:8580378).<br>This process leads to activation of several pathways including STAT, phosphoinositide-3-kinase/PI3K and mitogen-activated protein kinase/MAPK pathways (PubMed:25142963).<br>Functions as a T-cell growth factor and can increase NK-cell cytolytic activity as well (PubMed:6608729).<br>Promotes strong proliferation of activated B-cells and subsequently immunoglobulin production (PubMed:6438535).<br>Plays a pivotal role in regulating the adaptive immune system by controlling the survival and proliferation of regulatory T-cells, which are required for the maintenance of immune tolerance.<br>Moreover, participates in the differentiation and homeostasis of effector T-cell subsets, including Th1, Th2, Th17 as well as memory CD8-positive T-cells	P60568	17,628	153

#	Human Autoantigen Gene	Protein	Biological function	Acc. Number	Molecular weight (Da)	Length (Amino acids)
5	*MB*	Myoglobin	Monomeric heme protein which primary function is to store oxygen and facilitate its diffusion within muscle tissues. Reversibly binds oxygen through a pentacoordinated heme iron and enables its timely and efficient release as needed during periods of heightened demand (PubMed:30918256, PubMed:34679218). Depending on the oxidative conditions of tissues and cells, and in addition to its ability to bind oxygen, it also has a nitrite reductase activity whereby it regulates the production of bioactive nitric oxide (PubMed:32891753). Under stress conditions, like hypoxia and anoxia, it also protects cells against reactive oxygen species thanks to its pseudoperoxidase activity (PubMed:34679218).	P02144	17,184	154
6	*MBP*	Myelin basic protein	The classic group of MBP isoforms (isoform 4-isoform 14) are with PLP the most abundant protein components of the myelin membrane in the CNS. They have a role in both its formation and stabilization. The smaller isoforms might have an important role in remyelination of denuded axons in multiple sclerosis. The non-classic group of MBP isoforms (isoform 1-isoform 3/Golli-MBPs) may preferentially have a role in the early developing brain long before myelination, maybe as components of transcriptional complexes, and may also be involved in signaling pathways in T-cells and neural cells. Differential splicing events combined with optional post-translational modifications give a wide spectrum of isomers, with each of them potentially having a specialized function. Induces T-cell proliferation (PubMed: PMID: 8544862).	P02686	33,117	304

#	Human Autoantigen Gene	Protein	Biological function	Acc. Number	Molecular weight (Da)	Length (Amino acids)
7	*MMP2*	72 kDa type IV collagenase	Ubiquitinous metalloproteinase that is involved in diverse functions such as remodeling of the vasculature, angiogenesis, tissue repair, tumor invasion, inflammation, and atherosclerotic plaque rupture. As well as degrading extracellular matrix proteins, can also act on several nonmatrix proteins such as big endothelial 1 and beta-type CGRP promoting vasoconstriction. Also cleaves KISS at a Gly-|-Leu bond. Appears to have a role in myocardial cell death pathways. Contributes to myocardial oxidative stress by regulating the activity of GSK3beta. Cleaves GSK3beta in vitro. Involved in the formation of the fibrovascular tissues in association with MMP14. PEX, the C-terminal non-catalytic fragment of MMP2, posseses anti-angiogenic and anti-tumor properties and inhibits cell migration and cell adhesion to FGF2 and vitronectin. Ligand for integrinv/beta3 on the surface of blood vessels.	P08253	73,882	660

#	Human Autoantigen Gene	Protein	Biological function	Acc. Number	Molecular weight (Da)	Length (Amino acids)
8	*NGF*	Beta-nerve growth factor	Nerve growth factor is important for the development and maintenance of the sympathetic and sensory nervous systems (PubMed:14976160, PubMed:20978020). Extracellular ligand for the NTRK1 and NGFR receptors, activates cellular signaling cascades to regulate neuronal proliferation, differentiation and survival (Probable) (PubMed:20978020). The immature NGF precursor (proNGF) functions as a ligand for the heterodimeric receptor formed by SORCS2 and NGFR, and activates cellular signaling cascades that lead to inactivation of RAC1 and/or RAC2, reorganization of the actin cytoskeleton and neuronal growth cone collapse. In contrast to mature NGF, the precursor form (proNGF) promotes neuronal apoptosis (in vitro) (By similarity). Inhibits metalloproteinase-dependent proteolysis of platelet glycoprotein VI (PubMed:20164177). Binds lys un ophosphatidylinositol and lysophosphatidylserine between the two chains of the homodimer. The lipid-bound form promotes histamine relase from mast cells, contrary to the lipid-free form (By similarity).	P01138	26,959	241
9	*PRL*	Prolactin	Prolactin acts primarily on the mammary gland by promoting lactation.	P01236	25,876	227
10	*TPO*	Thyroid peroxidase	Iodination and coupling of the hormonogenic tyrosines in thyroglobulin to yield the thyroid hormones T3 and T4.	P07202	102,963	933
11	*GAD65*	Glutamate decarboxylase 2	Catalyzes the production of GABA (PubMed: 8999827)	Q9UGI5	47,344	419
12	*DPYD1*	Dihydropyrimi dine	Involved in pyrimidine base degradation (PubMed:1512248). Catalyzes the reduction of uracil and thymine (PubMed:1512248).	Q12882	111,401	1,025

#	Human Autoantigen Gene	Protein	Biological function	Acc. Number	Molecular weight (Da)	Length (Amino acids)
		dehydrogenase [NADP(+)]	Also involved the degradation of the chemotherapeutic drug 5-fluorouracil (PubMed:1512248)			
13	*ARE1a*	t-SNARE domain containing 1	-	A0AVG3	56,077	514
14	*D1L1a*	Mitotic spindle assembly checkpoint protein MAD1	Component of the spindle-assembly checkpoint that prevents the onset of anaphase until all chromosomes are properly aligned at the metaphase plate (PubMed:10049595, PubMed:20133940, PubMed:29162720).<br>Forms a heterotetrameric complex with the closed conformation form of MAD2L1 (C-MAD2) at unattached kinetochores during prometaphase, recruits an open conformation of MAD2L1 (O-MAD2) and promotes the conversion of O-MAD2 to C-MAD2, which ensures mitotic checkpoint signaling (PubMed:29162720).	Q9Y6D9	83,067	718
15	*F804A-1*	Zinc finger protein 804A	-	Q7Z570	136,888	1,209
16	*TCF4a*	Transcription factor 4	Transcription factor that binds to the immunoglobulin enhancer Mu-E5/KE5-motif. Involved in the initiation of neuronal differentiation. Activates transcription by binding to the E box (5'-CANNTG-3'). Binds to the E-box present in the somatostatin receptor 2 initiator element (SSTR2-INR) to activate transcription (By similarity).<br>Preferentially binds to either 5'-ACANNTGT-3' or 5'-CCANNTGG-3'	P15884	71,308	667
17	*SB2-1*	Transcription factor 4	Transcription factor that binds to the immunoglobulin enhancer Mu-E5/KE5-motif. Involved in the initiation of neuronal differentiation. Activates transcription by binding to the E box (5'-	P15884	71,308	667

#	Human Autoantigen Gene	Protein	Biological function	Acc. Number	Molecular weight (Da)	Length (Amino acids)
		(Different sequence)	CANNTG-3'). Binds to the E-box present in the somatostatin receptor 2 initiator element (SSTR2-INR) to activate transcription (By similarity).<br>Preferentially binds to either 5'-ACANNTGT-3' or 5'-CCANNTGG-3'			
18	*VRK2a*	Serine/threonine-protein kinase VRK2	Serine/threonine kinase that regulates several signal transduction pathways (PubMed:14645249, PubMed:16495336, PubMed:16704422, PubMed:17709393, PubMed:18286207, PubMed:18617507, PubMed:20679487).<br>Isoform 1 modulates the stress response to hypoxia and cytokines, such as interleukin-1 beta (IL1B) and this is dependent on its interaction with MAPK8IP1, which assembles mitogen-activated protein kinase (MAPK) complexes (PubMed:17709393).<br>Inhibition of signal transmission mediated by the assembly of MAPK8IP1-MAPK complexes reduces JNK phosphorylation and JUN-dependent transcription (PubMed:18286207).<br>Phosphorylates 'Thr-18' of p53/TP53, histone H3, and may also phosphorylate MAPK8IP1 (PubMed:16704422).<br>Phosphorylates BANF1 and disrupts its ability to bind DNA and reduces its binding to LEM domain-containing proteins (PubMed:16495336).<br>Down-regulates the transactivation of transcription induced by ERBB2, HRAS, BRAF, and MEK1 (PubMed:20679487).<br>Blocks the phosphorylation of ERK in response to ERBB2 and HRAS (PubMed:20679487).<br>Can also phosphorylate the following substrates that are commonly used to establish in vitro kinase activity: casein, MBP and histone H2B, but it is not sure that this is physiologically relevant (PubMed:14645249).	Q86Y07	58,141	508

#	Human Autoantigen Gene	Protein	Biological function	Acc. Number	Molecular weight (Da)	Length (Amino acids)
19	*DRD2a*	D(2) dopamine receptor	Dopamine receptor whose activity is mediated by G proteins which inhibit adenylyl cyclase (PubMed:21645528). Positively regulates postnatal regression of retinal hyaloid vessels via suppression of VEGFR2/KDR activity, downstream of OPN5 (By similarity).	P14416	50,619	443
20	*MP16-2*	Matrix metalloproteinase-16	Endopeptidase that degrades various components of the extracellular matrix, such as collagen type III and fibronectin. Activates progelatinase A. Involved in the matrix remodeling of blood vessels. Isoform short cleaves fibronectin and also collagen type III, but at lower rate. It has no effect on type I, II, IV and V collagen. However, upon interaction with CSPG4, it may be involved in degradation and invasion of type I collagen by melanoma cells (PubMed: 11278606)	P51512	69,521	607
21	*SB2-3*	TPR and ankyrin repeat-containing protein 1	-	O15050	336,221	2,925
22	*GABA$_A$Rα1*	Gamma-aminobutyric acid receptor subunit alpha-1	Alpha subunit of the heteropentameric ligand-gated chloride channel gated by Gamma-aminobutyric acid (GABA), a major inhibitory neurotransmitter in the brain (PubMed:23909897, PubMed:25489750, PubMed:29950725, PubMed:30602789). GABA-gated chloride channels, also named GABA(A) receptors (GABAAR), consist of five subunits arranged around a central pore and contain GABA active binding site(s) located at the alpha and beta subunit interface(s) (PubMed:29950725, PubMed:30602789). When activated by GABA, GABAARs selectively allow the flow of chloride anions across the cell membrane down their	P14867	51,802	456

#	Human Autoantigen Gene	Protein	Biological function	Acc. Number	Molecular weight (Da)	Length (Amino acids)
			electrochemical gradient (PubMed:23909897, PubMed:29950725, PubMed:30602789). Alpha-1/GABRA1-containing GABAARs are largely synaptic (By similarity). Chloride influx into the postsynaptic neuron following GABAAR opening decreases the neuron ability to generate a new action potential, thereby reducing nerve transmission (By similarity). GABAARs containing alpha-1 and beta-2 or -3 subunits exhibit synaptogenic activity; the gamma-2 subunit being necessary but not sufficient to induce rapid synaptic contacts formation (PubMed:23909897, PubMed:25489750). GABAARs function also as histamine receptor where histamine binds at the interface of two neighboring beta subunits and potentiates GABA response (By similarity). GABAARs containing alpha, beta and epsilon subunits also permit spontaneous chloride channel activity while preserving the structural information required for GABA-gated openings (By similarity). Alpha-1-mediated plasticity in the orbitofrontal cortex regulates context-dependent action selection (By similarity). Together with rho subunits, may also control neuronal and glial GABAergic transmission in the cerebellum (By similarity).			
23	*NMDAR*	Glutamate receptor ionotropic, NMDA 1	Component of NMDA receptor complexes that function as heterotetrameric, ligand-gated ion channels with high calcium permeability and voltage-dependent sensitivity to magnesium. Channel activation requires binding of the neurotransmitter glutamate to the epsilon subunit, glycine binding to the zeta subunit, plus membrane depolarization to eliminate channel	Q05586	105,373	938

#	Human Autoantigen Gene	Protein	Biological function	Acc. Number	Molecular weight (Da)	Length (Amino acids)
			inhibition by Mg^{2+} (PubMed:26875626, PubMed:26919761, PubMed:28105280, PubMed:28126851, PubMed:7685113). Sensitivity to glutamate and channel kinetics depend on the subunit composition (PubMed:26919761).			
24	*PDHA1*	Pyruvate dehydrogenase E1 component subunit alpha, somatic form, mitochondrial	The pyruvate dehydrogenase complex catalyzes the overall conversion of pyruvate to acetyl-CoA and CO_2, and thereby links the glycolytic pathway to the tricarboxylic cycle (PubMed:17474719).	P08559	43,296	390
25	*CA1Ca*	Voltage-dependent calcium channel subunit alpha-2/delta-1	The alpha-2/delta subunit of voltage-dependent calcium channels regulates calcium current density and activation/inactivation kinetics of the calcium channel (PubMed:35293990). Plays an important role in excitation-contraction coupling (By similarity)	P54289	124,568	1,103
26	*SLC39a*	Zinc transporter ZIP9	Transports zinc ions across cell and organelle membranes into the cytoplasm and regulates intracellular zinc homeostasis (PubMed:19420709, PubMed:25014355, PubMed:28219737). Participates in the zinc ions efflux out of the secretory compartments (PubMed:19420709). Regulates intracellular zinc level, resulting in the enhancement of AKT1 and MAPK3/MAPK1 (Erk1/2) phosphorylation in response to the BCR activation (PubMed:23505453). Also functions as a membrane androgen receptor that mediates, through a G protein, the non-classical androgen signaling pathway, characterized by the activation of MAPK3/MAPK1 (Erk1/2) and transcription factors CREB1 or ATF1 (By similarity).	Q9NUM 3	32,251	307

#	Human Autoantigen Gene	Protein	Biological function	Acc. Number	Molecular weight (Da)	Length (Amino acids)
			This pathway contributes to CLDN1 and CLDN5 expression and tight junction formation between adjacent Sertoli cells (By similarity).<br>Mediates androgen-induced vascular endothelial cell proliferation through activation of an inhibitory G protein leading to the AKT1 and MAPK3/MAPK1 (Erk1/2) activation which in turn modulate inhibition (phosphorylation) of GSK3B and CCND1 transcription (PubMed:34555425).<br>Moreover, has dual functions as a membrane-bound androgen receptor and as an androgen-dependent zinc transporter both of which are mediated through an inhibitory G protein (Gi) that mediates both MAP kinase and zinc signaling leading to the androgen-dependent apoptotic process (PubMed:25014355, PubMed:28219737).			
27	*ERC4a*	DNA repair endonuclease XPF	Catalytic component of a structure-specific DNA repair endonuclease responsible for the 5-prime incision during DNA repair, and which is essential for nucleotide excision repair (NER) and interstrand cross-link (ICL) repair (PubMed: 10413517)	Q92889	104,486	916
28	*F9B1*	Protein turtle homolog B	Transmembrane protein which is abundantly expressed in interneurons, where it may regulate inhibitory synapse development. May mediate homophilic cell adhesion.	Q9UPX0	147,089	1,349
29	*NRGN-1*	Neurogranin	Acts as a 'third messenger' substrate of protein kinase C-mediated molecular cascades during synaptic development and remodeling. Binds to calmodulin in the absence of calcium (By similarity).	Q92686	7,618	78
30	*SB2-2*	TPR and ankyrin repeat-containing	Antigen recognition by cytotoxic T lymphocytes	O15050	336,221	2,925

#	Human Autoantigen Gene	Protein	Biological function	Acc. Number	Molecular weight (Da)	Length (Amino acids)
		protein 1 (Different sequence)				

Supplementary Table S2: Distribution of *Toxoplasma gondii* homolog proteins identified in this study across clonal and atypical/recombinant genotypes.

Human Autoantigen	*T. gondii* Homolog Protein	Accession	Strain	Genotype Classification
ENO2	Enolase 1	XP_002365578	ME49	Type II (clonal)
ENO2	Enolase 2	XP_002365579	ME49	Type II (clonal)
ENO2	PGAM2	Q9BPL7	ME49	Type II (clonal)
TPO	Calcium-binding EGF protein	KFG44949	FOU	Atypical
TPO	Calcium-binding EGF protein	KYK67774	TgCatPRC2	Recombinant
TPO	Calcium-binding EGF protein	KFG63764	RUB	Atypical
TPO	Calcium-binding EGF protein	CEL74380	VEG	Type III (clonal)
GAD65	Protein kinase (incomplete catalytic triad)	XP_018637623.1	ME49	Type II (clonal)
GAD65	Putative helicase (partial)	PUA92631.1	ME49	Type II (clonal)
VRK2	Casein kinase	RQX72942	CAST	Atypical
VRK2	Casein kinase	PIM01748	COUG	Recombinant
VRK2	Casein kinase	CEL78736	VEG	Type III (clonal)
